# Development of Targeted Protein-Displaying Technology with a Novel Carbon Material

**DOI:** 10.3390/biotech12010002

**Published:** 2022-12-25

**Authors:** Akihito Nakanishi, Naotaka Yamamoto, Yuri Sakihama, Tomoya Okino, Naoki Matoba

**Affiliations:** 1 Graduate School of Bionics, Tokyo University of Technology, Tokyo 192-0982, Japan; 2 School of Bioscience and Biotechnology, Tokyo University of Technology, Tokyo 192-0982, Japan; 3 College of Science and Engineering, Aoyama Gakuin University, Sagamihara 252-5258, Japan; 4 Kansai Coke & Chemicals Co., Ltd., Amagasaki 661-0976, Japan

**Keywords:** carbon material, avidin-biotin binding, protein-displaying carbon material

## Abstract

This study reports a new carbon material and its specific display of targeted protein. The properties of the carbon materials fabricated with carbon black MOGUL^®^ were analyzed. The carbon materials were spherical structures with 55.421 µm as a median value. The specific surface area, pore volume, average pore diameter, and total of the acidic functional group were 130 m^2^·g^−1^, 0.55 cm^3^·g^−1^, 17.2 nm, and 0.29 mEq·g^−1^, respectively. The adsorption–desorption isoform of the carbon materials showed type IV of the hysteresis loop as defined by IUPAC, indicating non-uniform mesoporous structures (2–50 nm). The distribution of the log differential pore volume also indicated non-uniform porous structures because (i) the difference between the average pore size and the most frequent pore size was significant and (ii) the σ value was larger than the average value regarding the pore sizes. However, 10–90% of the integrated values of the log differential pore volume were 57.4% of the total integrated values, and the distribution was similar to the Gauss distribution model. Although the value of the total of the acidic functional group was 2.5–5.4 times lower than the values of the HPLC columns, the carbon materials require good scaffold quality rather than good HPLC quality. Therefore, the amounts could be enough for the scaffold of biotin hydrazide. To demonstrate the property of displaying the targeted proteins, carbon materials displaying biotin hydrazide by covalent bonding were prepared and avidin-labeled horse radish peroxidase (HRP) was bound to the biotin region. The carbon materials were porous structures, so the unspecific adsorption of HRP was estimated. Then, the maintenance ratios of HRP activities were analyzed in the repeated-use-with-wash processes after each evaluation, resulting in the activities of HRP on the carbon materials being treated with biotin hydrazide being significantly maintained compared to that of the ones without biotin hydrazide. The study revealed the properties of the carbon materials and indicated the display of HRP, suggesting that the carbon materials could be a new material for displaying targeted proteins.

## 1. Introduction

Protein provides functional effects in various fields, such as industry and research [1,2]. Enzymes especially are used due to their high activity and safety in a wide range of these fields, such as the food [3], biomedical [4], and cosmetic industries [5], among others. Enzymes are useful but normally expensive. Therefore, although the reuse of value-added enzymes shows economical and convenient merits, reuse faces difficulties concerning separation and recovery as most enzymes have the property of water solubility. The selective recovery of enzymes from the aqueous layer requires complicated processes and costs, such that the development of an easy and low-cost technique to reuse enzymes is needed. To date, technological developments to reuse enzymes have advanced in various fields, resulting in the easy collection methods of protein-displaying scaffolds with sedimentation by their own weight or low-speed centrifugation. So far, enzymes have been displayed on glass beads [6,7], magnetic beads [8], and yeast cell surfaces [9,10]. However, the enzyme-collecting methods with glass and magnet are normally expensive at 667 USD·kg^−1^ (Sigma-Aldrich, St. Louis, MO, USA) and 221 USD·mL^−1^ (Recenttec, Tokyo, Japan) in 2022. Additionally, the method involving the cell-surface engineering of yeast potentially contains the problem of gene-modified organisms. Therefore, the development of an enzyme-collecting method that overcomes these problems is needed.

**Figure 1:**
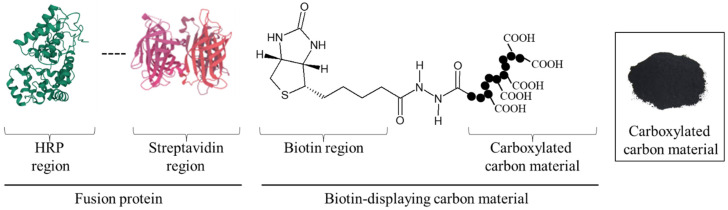
Concept of targeted protein-displaying system on carbon material.

Coal has long been an important energy resource to support industry. As coal can be used not only as a fuel but also as a low-cost source of carbon powder at a few USD·kg^−1^, to date it has been greatly in-demand. Moreover, adding the values of carbon powder provides a new method of using carbon materials for society. Therefore, with the granulation technology of carbon powder, a new ‘carbon material’ was prepared in this research. The carbon material can be easily collected with sedimentation either by its own weight or low-speed centrifugation. The property of the sediments conceptually suits enzyme reuse so that the availability of the carbon materials is remarkably improved, selectively displaying enzymes on the carbon materials. To develop a technique for displaying the targeted enzyme on carbon materials and to demonstrate the reuse of the displaying enzyme, in this study the construction of the technology needed to set up the scaffold for enzyme display on the surface of the carbon materials was tried based on chemical and biological techniques (Figure 1). Firstly, carboxylation on the surface of carbon materials was attempted. The carboxylated carbon materials were fabricated with carbon powder treated by hypochlorite to carboxylate its surface. Secondly, the biotin display connecting to the carboxy group on carbon materials was purposed. In biotechnology, hydrazide groups are aldehyde-reactive chemical groups used as biomolecular probes that crosslink and label carbonyls such as proteins and sugars [11]. Additionally, the interaction of biotin with avidin has been extensively used for the detection and purification of specific proteins [12,13]. The avidin–biotin interaction has been used for highly sensitive evaluation since this interaction is highly stable (dissociation constant K_D_ = 10^−15^ M), and as the interacting part is small in size and hardly interferes with the functionality of the labeled molecule [14]. The hydrazide group of biotin hydrazide was reacted with the carboxy group on the surface of the carbon materials as a cross-linking target, and biotin display on the carbon materials was tried. Finally, the specific display of avidin-fused enzymes targeting biotin on the carbon materials was achieved. To evaluate the construction of the enzyme-displaying system on the carbon materials, streptavidin-fused horseradish peroxidase (HRP), universally used as the labelling enzyme on SDS-PAGE [15], was used because of its stability and high reaction activity.

In this study, the HRP-displaying carbon materials were prepared by fabricating the carboxylated carbon materials, crosslinking biotin hydrazide with the carboxyl group as an aldehyde-reactive chemical group, and performing the avidin–biotin interaction. Additionally, after treatment with avidin-fused HRP, the carbon materials were evaluated for the display of the targeted proteins, their reuse was demonstrated. This is the first report to provide the value of targeted protein display on carbon materials fabricated with carbon powder for waste and reuse of enzymes displayed on the carbon materials.

## 2. Materials and Methods

### 2.1. Preparation of Carbon Material

Carbon black MOGUL^®^ (Cabot, Boston, MA, USA) was dispersed in 0.85% polyvinyl alcohol (PVA500) (Kanto Chemical, Tokyo, Japan). The treated carbon black was spray-dried, and the sphered CB was prepared with the dried materials with a tube-shaped furnace of nitrogen at 800 °C. The sphered CB was adjusted to 10% (w/v) into 5% nitric acid and boiled for 60 min. The prepared sphered CB was collected with a suction filter, washed with water at 60 °C, and then dried at 115 °C for over 24 h. Finally, the carbon materials were obtained. 

### 2.2. Analyses for Structural Properties of Carbon Material

The particle size distribution of carbon materials was analyzed with a laser diffraction particle size distribution analyzer SALD-2000A (Shimadzu, Kyoto, Japan). 

Approximately 100 mg of the carbon materials was evaluated under the following conditions: the carbon materials were dispersed in distilled water with surfactant, and the refractive index parameter of the instrument was measured at 1.7–0.2. The aspect ratio of carbon materials was evaluated by measuring 1000 particles of carbon materials with a scanning electron microscope (SEM) (JSM-6060LV: Japan Electron Optics Laboratory Co., Ltd., Tokyo, Japan). Before scanning, carbon materials were coated with Au particles with an ion coater (IB-2: Eiko Engineering, Tokyo, Japan). A total of 1000 particles of Au particle-coated carbon material was observed for measuring the sizes with SEM. The properties of the porous structure of the carbon materials, including the specific surface area (m^2^·g^−1^), pore volume (cm^3^·g^−1^), and pore diameter (nm), were determined by nitrogen adsorption isotherms with BELSORP-mini (Microtrac Bell, Osaka, Japan). After the preparation of about 50 mg of the carbon materials under a vacuum state at 250 °C for 180 min, nitrogen adsorption isotherms of the carbon materials were obtained at −196 °C with liquid nitrogen. With the nitrogen adsorption isotherms, the specific surface area and total pore volume were determined by the BET method [16], and the pore diameters were detected by the BJH method [17]. Additionally, the number of acidic functional groups was quantified by Boehm methods [18]. An amount of 2 mg of the powdered carbon was stirred in 0.1 M NaOC_2_H_5_ for 2 h, left for 24 h, and filtered to collect the supernatant. To evaluate the total number of acidic functional groups, the residue of NaOC_2_H_5_ in the filtered supernatant was determined with 0.1 M HCl as a titrimetric analysis. 

### 2.3. Analysis for the Activity of Horseradish Peroxidase on Carbon Material

A measure of 10 mg of carbon materials was added into 1000 µL of each solution optimized in this study as follows: (1) DMSO adjusted to 1.3 mM biotin hydrazide; (2) DMSO adjusted to 1.3 mM biotin hydrazide and 240 mM cyanamide; (3) DMSO and 240 mM cyanamide [19,20,21]. Each reaction tube containing carbon material was set on the Microtube Rotator MTR-103 (ASONE, Osaka, Japan) at 20 rpm for 2 h at 23 °C. After the reaction treatment, the reaction tube was centrifuged to collect the carbon materials (15,000× *g*, 1 min, 23 °C), and then the supernatant was discarded. The treated carbon materials were washed with 1 mL of PBS twice. The washed carbon materials were immersed in 1 mL of PBS containing horseradish peroxidase (HRP)-labeled streptavidin (Proteintech Rosemont, Group Inc., IL, USA) at a dilution rate of 1:1000. The treatment was performed with a Microtube Rotator MTR-103 at 20 rpm for 5 min at 23 °C. The treated carbon materials were washed twice in the manner mentioned above and introduced into 1 mL of PBS-based reaction buffer containing 3,3′,5,5′-tetramethyl-benzidene (TMB) (Funakoshi, Tokyo, Japan) at a dilution ratio of 1:10. The absorbance range of TMB is around 285 nm as a peak. The charge transfer complex was obtained after the oxidization of TMB, and the complex shifted the absorbance range to around 650 nm as a peak. According to Lambert–Beer’s law, the HRP-oxidation activity was evaluated by comparing the concentrations of the complex, indicating the absorbances at 650 nm. For the repeated use of the carbon materials, the supernatant was replaced with the new PBS-based reaction buffer after two washes. The activity maintenance ratio upon second use was calculated as the ratio of the absorbance value of the second use at 650 nm versus the one of first use at 650 nm. The activity maintenance ratio at third use was also calculated as the ratio of the absorbance value of the third use at 650 nm versus the one of first use at 650 nm. 

## 3. Results

### 3.1. Properties of Carbon Material

**Figure 2:**
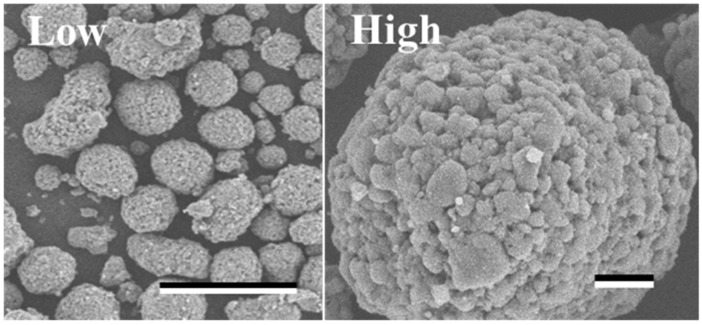
SEM image of the carbon materials.

**Figure 3:**
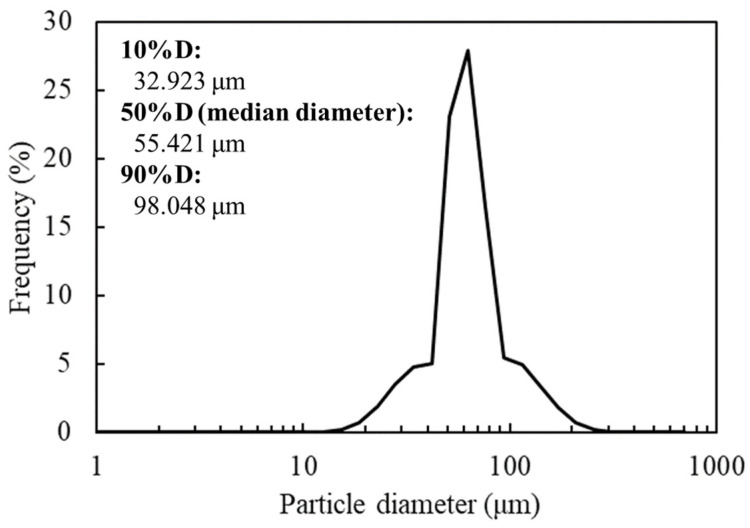
Size distribution of the carbon materials.

**Figure 4:**
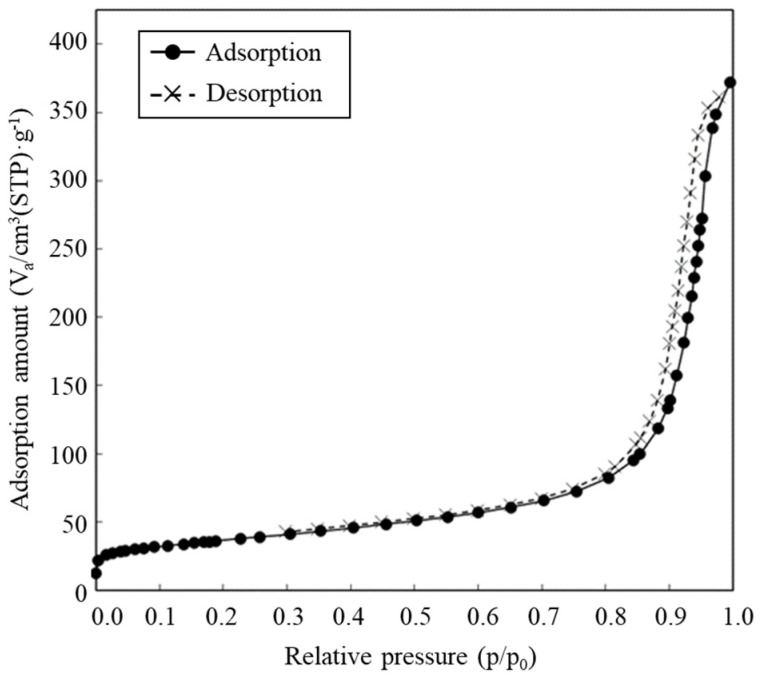
Adsorption-desorption isotherms.

**Figure 5:**
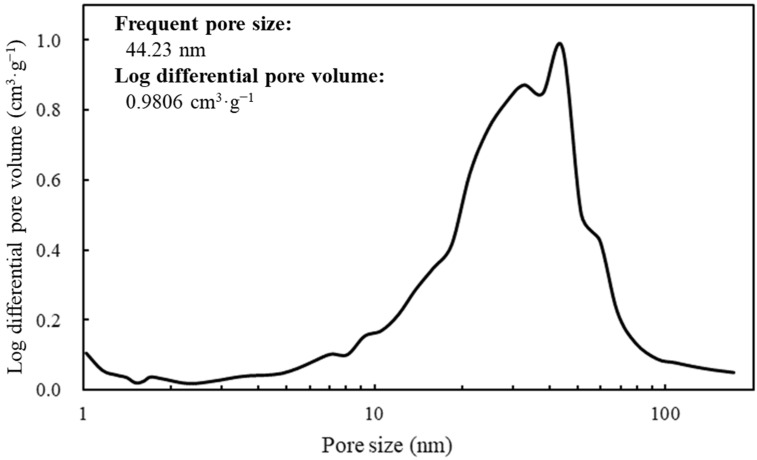
Log differential pore volume at each pore size.

The SEM image revealed the spherical structure of the carbon materials visually (Figure 2). According to the analysis of 1000 particles of carbon materials in SEM images, the particles showed 1.1 ± 0.1 of the aspect ratio so that the carbon materials were slightly elongated spherical structures. In addition, non-uniformity in the sizes of the structures was also revealed. The particle sizes of the carbon materials were investigated with a laser diffraction particle size analyzer to reveal the particle size (Figure 3). Analysis of the cumulative distribution of the particle size frequency showed that the particle sizes at 10%, 50% (a median diameter), and 90% of the cumulative distribution were 32.923 µm, 55.421 µm, and 98.048 µm, respectively. While 80% of the appearance frequency of the particles of the carbon materials was in the range of 32.923–98.048 µm, the lower limit of 0.03 to 20 µm and the upper limit of 170 to 700 µm were included in less than 1% at each fraction. Additionally, the properties of the carbon materials were evaluated in terms of material engineering and chemistry (Table 1). In terms of the material engineering properties of the carbon materials, the specific surface area, pore volume, and averaged pore diameter of the carbon materials and MOGUL were 130 m^2^·g^−1^ and 128 m^2^·g^−1^, 0.55 cm^3^·g^−1^ and 0.69 cm^3^·g^−1^, and 17.2 nm and 22.1 nm, respectively. On the other hand, in terms of chemistry, the whole amount of acidic functional group per gram of carbon material and MOGUL were 0.29 mEq·g^−1^ and 0.51 mEq·g^−1^, respectively. To survey the presence and sizes of the pores of the carbon materials, the data of the adsorption amount were plotted related to the relative pressure as the analysis of the adsorption–desorption isotherm (Figure 4). Although the plot patterns in the adsorption and desorption isotherms did not exhibit significant differences, the plot patterns showed a hysteresis loop with the closing point. An analysis of the logarithmic differential pore volume distribution was conducted to reveal the distribution of pore sizes in the carbon materials (Figure 5). The frequent pore size was 44.23 nm, and the log differential pore volume was 0.9806 cm^3^·g^−1^. However, regarding the frequent pore size, the ratio of the log differential pore volume at the frequent pore size was only 10.8% of the whole log differential pore volume. Additionally, the average and standard deviation of the log differential pore volume of all the carbon materials were 0.232 cm^3^·g^−1^ and 0.282 cm^3^·g^−1^, respectively. On the other hand, in 10–90% of the cumulative distribution of the log differential pore volume, the average and standard deviation were 0.558 cm^3^·g^−1^ and 0.273 cm^3^·g^−1^, respectively.

Scale-bars were 100 µm and 10 µm under low (×350) and high (×1600) magnifications.

The frequency of the particle sizes of the carbon materials was shown by spectrum. The expressions 10%D, 50%D (median diameter), and 90%D mean 10%, 50% and 90% of the cumulative distribution of the carbon materials.

The values of adsorption and desorption were plotted against the relative pressure as circle and cross marks, respectively. 

The spectrum of the values of the log differential pore volume is shown against pore sizes. 

### 3.2. HRP-Activity on Carbon Material

**Figure 6:**
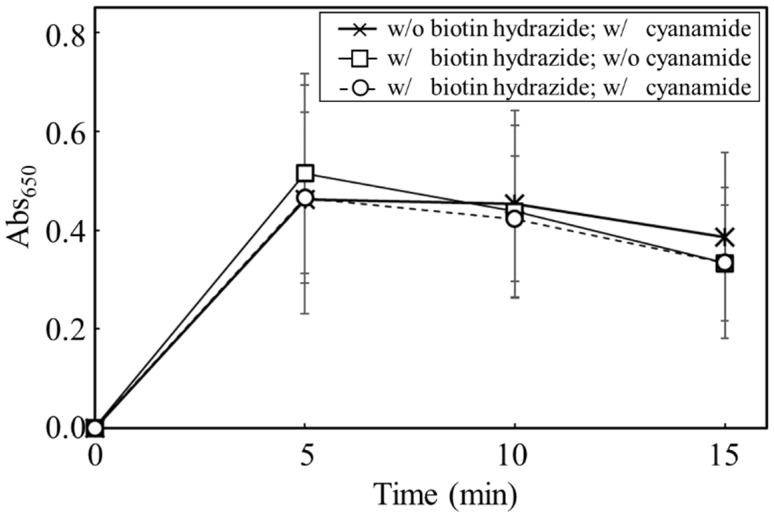
Time-course profiling of Abs_650_ for the carbon materials prepared with each treatment.

**Figure 7:**
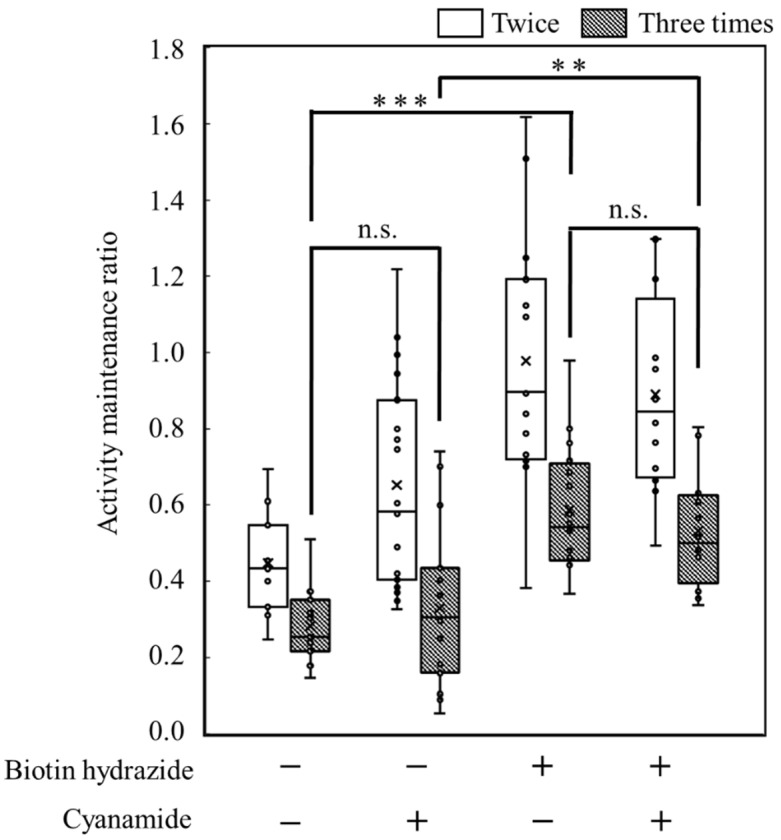
Box plot of the activity maintenance ratio for repeated use of the carbon materials prepared with each treatment. **, *** and n.s. indicated *p* < 0.01, *p* < 0.001 and not significant respectively.

The objective of this study was to prove the display of the targeted proteins via biotin hydrazide on the carbon materials. Therefore, by measuring the activity of HRP fused with avidin, the display of the targeted protein was attempted (Figure 6). To analyze the display of HRP on the carbon materials, the activities of HRP on the carbon materials prepared by each treatment (w/o biotin hydrazide, w/cyanamide; w/biotin hydrazide, w/o cyanamide; w/biotin hydrazide, w/cyanamide) were evaluated. All the reactions by HRP on the carbon materials prepared in all conditions reached a plateau within 5 min, including the reaction by the HRP-carbon materials prepared without biotin hydrazide. In addition, a huge distribution was detected at every time point under all conditions, and the standard deviations were almost stacked. This study proposed carbon materials regarding the unspecific adhesion and the display of HRP as follows: the unspecifically adsorbed HRP activity could gradually decrease due to peeling off by repeated use; on the other hand, HRP specifically displayed on the carbon materials via biotin could maintain its activity even after repeat use. The maintenance ratio of HRP activity was analyzed for repeated use to evaluate the specific display of HRP via biotin on the carbon materials with a box plot (Figure 7). In the second use, the maintenance ratios of HRP activities on each carbon material (w/o biotin hydrazide, w/o cyanamide; w/o biotin hydrazide, w/cyanamide; w/biotin hydrazide, w/o cyanamide; w/biotin hydrazide, w/cyanamide) decreased to 0.45 ± 0.13, 0.65 ± 0.27, 0.98 ± 0.33 and 0.89 ± 0.26, respectively; from the second to the third use, 0.28 ± 0.10, 0.33 ± 0.20, 0.59 ± 0.17 and 0.53 ± 0.15, respectively. This means that all the activities of HRP were depressed with all carbon materials prepared by each HRP-displaying treatment. In particular, the maintenance ratios of HRP activities in the second and third uses were analyzed by two-sample *t*-test, and the results were *p* = 0.0040, *p* = 0.00021, *p* = 0.00030, and *p* = 0.00073, respectively, indicating that the statistically significant decrement depended on *p* < 0.01 **. Regarding the HRP-activity maintenance ratios in the third use, (i) the HRP activity in the systems without cyanamide ([w/o biotin hydrazide, w/o cyanamide] versus [w/biotin hydrazide, w/o cyanamide]) and (ii) the HRP activity in the systems with cyanamide ([w/o biotin hydrazide, w/cyanamide] versus [w/biotin hydrazide, w/cyanamide]) were also analyzed by two-sample *t*-test. The results in systems of (i) and (ii) were *p* = 0.0000038 and *p* = 0.0036, respectively, meaning the significance levels were *p* < 0.001 *** and *p* < 0.01 **, respectively. The maintenance ratio of HRP activities of each biotin hydrazide system (w/biotin hydrazide, w/o cyanamide; w/biotin hydrazide, w/cyanamide) was also analyzed by two-sample *t*-test, and the result was *p* = 0.37.

An absorbance of 650 nm was measured to evaluate HRP activities on the carbon materials after preparation under different conditions of biotin hydrazide and cyanamide. The data was plotted by cross (w/o biotin hydrazide; w/cyanamide), square (w/biotin hydrazide; w/o cyanamide), and circle (w/biotin hydrazide; w/cyanamide). The values were averaged from 17-time, 16-time, and 12-time experiments (See Figure 7).

The activity maintenance ratio was calculated based on data of an absorbance of 650 nm with the carbon materials after preparation under different conditions of biotin hydrazide and cyanamide to display HRP. The values were averaged from 12−17-time experiments. In Figure 7, the significance levels are indicated by asterisks (**: *p* < 0.01 and ***: *p* < 0.001) and n.s. (not significant).

## 4. Discussion

In this study, a system to display streptavidin-labeled HRP on carbon materials via biotin hydrazide was proposed as a new protein-displaying system (Figure 1). The important point in our case was that biotin hydrazide should be bound with carboxylic acid groups on the carbon materials. Although it is difficult to get hydrazide to react with the carboxylic acid group, there are examples demonstrating the reaction. For instance, Tiba et al. performed the reactions of aliphatic carboxylic acid hydrazide and the carboxylic acid groups [22], and Sato conducted the reaction of hydrazide and adipic acid as the carboxylic acid group [23]. In detail, Tiba et al. performed the synthesis of N,N’-didecanoylhydrazine, phthalhydrazide, and N-decanoyl-N’-phthaloylhydrazine with the reaction of decanoyl hydrazide and phthalic acid [22]. The notable aspect was that N,N’-didecanoylhydrazine and N-decanoyl-N’-phthaloylhydrazine were successfully synthesized without adding acids and N,N’-didecanoylhydrazine was also synthesized without water. On the other hand, Sato developed the following method for synthesizing the adipic acid dihydrazide: 10 g of adipic acid is added to 18 mL of hydrazine hydrate 80%, and the mixture is stirred for 24 h. After this, 6.2 g of adipic acid dihydrazide is obtained by extraction by adding ethanol [23]. The important points in this case are the following: (i) the synthesis of adipic acid dihydrazide without adding the acids, and (ii) the progress of the reaction with a solvent such as water, which could not disturb the reaction. As an additional important point, Sato also showed that the reaction could be performed at room temperature. As mentioned above, in the case of carbon materials, the reaction of biotin hydrazide and the carboxyl group could occur in DMSO without adding acids at room temperature. According to the observation of SEM images (Figure 2), the carbon materials were almost spherical structures, as indicated by 1.1 ± 0.1 of the aspect ratio. The spherical structure of the carbon material means that it could hardly be broken structurally, implying that the carbon materials could withstand repeat use. The lack of size uniformity of the carbon materials was revealed by SEM imaging. To evaluate the size quality of the carbon material, the distribution of the particle sizes was analyzed with a laser diffraction particle size analyzer (Figure 3). To discuss the unspecific adsorption of proteins on the carbon materials, the specific surface area, pore volume, and average pore diameter were evaluated as properties of the carbon materials. Additionally, to display the biotin hydrazide on the carbon materials via a covalent bond, the entirety of the acidic functional group was assessed. The specific surface area and pore volume of the wood charcoals, such as oak and sawtooth oak, were 100–400 m^2^·g^−1^ and 0.1–1 cm^3^·g^−1^ [24], indicating that the carbon materials in this study should have a porous structure. Additionally, as shown in Table 1, the averaged pore diameters of the carbon material and MOGUL were 17.2 nm and 22.1 nm, respectively, defined as a mesopore structure [25,26]. On the other hand, the specific surface areas of the carbon material and MOGUL were 130 m^2^·g^−1^ and 128 m^2^·g^−1^, respectively; the pore volumes were 0.55 cm^3^·g^−1^ and 0.69 cm^3^·g^−1^. By the fabrication of the carbon materials with MOGUL as the raw material, the averaged pore diameter and pore volume decreased to 78% and 79%; on the other hand, the specific surface area only demonstrated a 2% increment. Thus, although differences in the average pore diameter and pore volume were detected by the MOGUL process, few differences in specific surface area were revealed. The porous sizes of the carbon materials were enough for the HRP display, considering 7.6 nm of pores necessary for storing HRP, as reported in [27]. The maintained surface areas of the carbon materials kept the characteristics detected by MOGUL because the surface area affects the amount of the protein display [28]. Therefore, the carbon materials were shown to be useful materials for HRP display, even after the MOGUL process. Additionally, sieve silicate represented 570–912 m^2^·g^−1^ of the specific surface area and 0.47–2.28 cm^3^·g^−1^ of the pore volume [28]; nonionic triblock copolymer represented 632.3–708.5 m^2^·g^−1^ of the specific surface area and 0.85–0.92 cm^3^·g^−1^ of the pore volume [27]. Compared to these data, although the specific surface areas of the carbon materials were low, from the point of view of physical properties, the pore volumes of the carbon materials were not too low. Comparing the sizes of the carbon materials with other particle sizes in the biological field, the 55.421 μm median diameter of the carbon materials was larger than the 4–10 μm values for glass bead particles used in flow cytometry but smaller than the 100–300 μm values of particles used for cell disruption [29]. The specific surface area and pore volume of the carbon materials shown in Table 1 were compared with those of wood charcoals as porous materials [30], and the specific surface area and pore volume of the charcoal were 446 m^2^·g^−1^ and 0.13 cm^3^·g^−1^. As the data show, the specific surface area and pore volume of the carbon materials were 3.4 times smaller and 4.2 times larger than those of charcoal. Although the specific surface area of the carbon materials was smaller than that of charcoal, the physical properties could be enough to display the protein, as shown above. The entirely of the acidic functional group of the carbon materials was 0.29 mEq·g^−1^, as shown in Table 1, and the ion exchange amounts of the fillers in the packed column for HPLC were 0.72–1.57 mEq·g^−1^ [31]. The values of the HPLC fillers were only 2.5–5.4 times higher than those of the carbon materials despite the HPLC quality, so the entirety of the acidic functional group of the carbon materials indicated sufficient binding for biotin hydrazide in this study. As the result of adsorption–desorption isotherm of the carbon material (Figure 4), the porous type of the carbon material was type IV, indicating the existence of mesopores (pores of 2–50 nm) according to the IUPAC isotherm classification [26]. Additionally, the shape was similar to the type H2 [26,32], indicating that the carbon material was the material with a less distinct pore size distribution and pore structure [26]. The hysteresis shape also indicated capillary condensation mainly in the mesopore region [26]. However, the adsorption–desorption isotherms could only predict the approximate porous structures without detail, so the distribution of the pore diameter and volume of the carbon materials, which are important properties of the materials, should be demonstrated to discuss the carbon materials in detail. To clarify the details of the hysteresis loop indicated by the adsorption–desorption isotherm analysis, the results of the logarithmic differential pore volume distribution were evaluated (Figure 5). As with the results of the adsorption–desorption isotherm, the result of the distribution of the logarithmic differential pore volume also showed the carbon material as the material with a less distinct pore size distribution and pore structure. In the analytical results of the log differential pore volume, 0.232 cm^3^·g^−1^ of the average log differential pore volume was significantly different from the 0.9806 cm^3^·g^−1^ for the log differential pore volume at 44.23 nm, as the high-frequency pore size. Additionally, 0.282 cm^3^·g^−1^, as the standard deviation of the log differential pore volume, was larger than 0.232 cm^3^·g^−1^, the average. Furthermore, the log differential pore volume at the high-frequency pore size occupied only 11% of the total volume, indicating a broad distribution. These results strongly indicate that the distribution was not a Gaussian distribution, statistically. However, in 10–90% of the cumulative distribution of the log differential pore volume, the average and standard deviation of the log differential pore volume were 0.558 cm^3^·g^−1^ and 0.273 cm^3^·g^−1^, respectively. The average value (0.558 cm^3^·g^−1^) of the log differential pore volume approached 0.9806 cm^3^·g^−1^ at 44.23 nm as the high-frequency pore size; the standard deviation value (0.273 cm^3^·g^−1^) was lower than the average value. Although, the integrated value could not reach 68.2%, 57.4% of the log differential pore volume was in the range of 0.286–0.831 cm^3^·g^−1^ defined by the σ range. These results significantly indicate that the distribution in 10–90% of the range of the log differential pore volume approached a Gaussian distribution, statistically. As shown above, the results reveal that the values of the log differential pore volumes were widely distributed. However, most proteins could access 5–80 nm of the pore sizes [27,28,33], so this study could use the pores of the carbon materials. However, the average and standard deviation in 10–90% of the cumulative distribution of the log differential pore volume were 0.558 cm^3^·g^−1^ and 0.273 cm^3^·g^−1^, respectively, and 57.4% of the log differential pore volume was in the range of 0.286–0.831 cm^3^·g^−1^, defined by the σ values. Although this integrated value did not reach 68.2% shown by the σ value, the results suggest that the distribution approached a broad Gaussian distribution. 

The possibility of displaying the targeted proteins on the carbon materials was evaluated by using HRP fused with avidin (Figure 6). The absorbances at 650 nm in all reaction conditions indicated the max values, meaning that the oxidization of TMB was completed in 5 min despite the reaction conditions, with or without biotin hydrazide and with or without cyanamide. From another viewpoint, the HRP activity on the carbon materials treated without biotin hydrazide showed no significant difference from the other activities. In fact, there are many reports of the unspecific adsorption of proteins on porous structures, such as nonionic triblock copolymer and sieve silicate [27,28]. Therefore, the results strongly indicate the unspecific adsorption of the avidin-labeled HRP on the carbon materials as the porous structure. As shown in Table 1, the average pore diameter of the carbon materials was 17.2 nm. Chen et al. stored the HRP with a pore size of 7.6 nm [27], meaning that HRP could easily access the porous structure of the carbon materials. Therefore, HRP on the carbon materials could possibly maintain the activities on the carbon materials by unspecific adsorption. The carbon materials treated with HRP were repeatedly used with wash after use to show the advantage of using biotin hydrazide for protein display on the carbon materials. The HRP activity making contact with the carbon materials via biotin hydrazide could be maintained; on the other hand, the activity without contact via biotin hydrazide decreased because of desorption. The activity-maintenance ratios of HRP in the repeated uses were evaluated to demonstrate the specific display of the specific proteins on carbon materials (Figure 7). The HRP activities under all conditions decreased under repeated use: the significance level decreased drastically (*p* < 0.001 ***) in the systems without biotin hydrazide (w/o biotin hydrazide, w/o cyanamide; w/o biotin hydrazide, w/cyanamide) and the systems with biotin hydrazide (w/biotin hydrazide, w/o cyanamide; w/biotin hydrazide, w/cyanamide), indicating that the nonspecific adsorption of HRP decreased. For the activity of HRP in the third use: (i) the HRP activity in the systems without cyanamide ([w/o biotin hydrazide, w/o cyanamide] and [w/biotin hydrazide, w/o cyanamide])) had significance levels of *p* < 0.001 ***; (ii) the HRP activity in the systems with cyanamide ([w/o biotin hydrazide, w/cyanamide] and [w/biotin hydrazide, w/cyanamide]) had significance levels of *p* < 0.01 **. Therefore, the maintenance of HRP on the carbon materials by using biotin hydrazide was statistically shown, and the facts could indicate a chemical bond between the carboxyl group on the carbon materials and the hydrazide group of biotin hydrazide. However, since there was no significant difference in HRP activity with or without cyanamide, cyanamide is not a useful mediator for amide bond formation of biotin hydrazide.

## 5. Conclusions

The properties of the carbon materials in this study were revealed to be as follows: a spherical and porous structure, a 55 µm median diameter, and a 17.2 nm average pore size. Additionally, the carboxylation of the carbon materials was also revealed, reaching 0.29 mEq·g^−1^, meaning that the carboxyl level was enough to display the biotin hydrazide with the filler data of the HPLC column. The results of the adsorption–desorption isotherm indicated type IV plotting, implying the existence of mesopores (2–50 nm) as the properties of the carbon materials. Additionally, the results of the adsorption–desorption isotherm drew an H2-like hysteresis loop, suggesting that the carbon materials had a less distinct distribution of pore sizes and pore structure. The analysis results of the logarithmic differential pore volume distribution also supported the suggestion of adsorption–desorption isotherms both because of the differences between the averaged value and the frequent value of pore sizes and the evaluation of the σ-value from the viewpoint of the Gauss distribution. However, in 10–90% of the cumulative distribution of the log differential pore volume, the ratio of the integrated values of the log differential pore volumes was 57.4% in the σ-range, indicating that the distribution could approach a Gaussian distribution. The purpose of this study was specifically to display the targeted proteins on carbon materials. Therefore, to show the display of the targeted proteins on the carbon materials via biotin hydrazide, the activity of HRP as the model of the targeted protein on the carbon materials was analyzed in each repeated use. The results showed that the maintenance ratio of the HRP activity on the carbon materials treated by biotin hydrazide was higher than that not treated by biotin hydrazide. This study indicates the effectiveness of a biological approach to displaying the targeted proteins on the carbon materials.

## Figures and Tables

**Table 1:** Properties of the carbon material.

Sample Name	Specific Surface Area (m^2^·g^−1^)	Pore Volume (cm^3^·g^−1^)	Average Pore Diameter (nm)	Whole Amount of Acidic Functional Group (mEq·g^−1^)
Carbon material	130 ± 13	0.55 ± 0.07	17.2 ± 2.4	0.29 ± 0.1
MOGUL (Raw material)	128 ± 13	0.69 ± 0.08	22.1 ± 3.1	0.51 ± 0.1

## Data Availability

Not applicable.
